# Duplication of the Pituitary Gland (DPG)-Plus Syndrome Associated With Midline Anomalies and Precocious Puberty: A Case Report and Review of the Literature

**DOI:** 10.3389/fendo.2021.685888

**Published:** 2021-05-26

**Authors:** Giovanni Prezioso, Maddalena Petraroli, Michela Bergonzani, Giusy Davino, Marialuisa Labate, Francesca Ormitti, Marilena Anghinoni, Enrico Sesenna, Susanna Esposito

**Affiliations:** ^1^ Pediatric Clinic, Department of Medicine and Surgery, University of Parma, Parma, Italy; ^2^ Maxillo-Facial Surgery Unit, Department of Medicine and Surgery, University of Parma, Parma, Italy; ^3^ Neuroradiology Unit, University Hospital of Parma, Parma, Italy

**Keywords:** pituitary, craniofacial abnormalities, midline malformations, pediatric endocrinology, precocious puberty

## Abstract

Duplication of the pituitary gland (DPG)-plus syndrome is a very rare developmental disorder with few cases described in the literature and characterized by multiple midline and central nervous system malformations. The hypothalamus and hypophysis involvement may be clinically associated with endocrine abnormalities. A 5.9-year-old female child was admitted to our Clinic for premature thelarche and acceleration of growth. DPG-plus syndrome with paired infundibula and pituitary glands was diagnosed after birth, when she appeared small for gestational age and she presented with lingual hypoplasia, cleft palate, right choanal stenosis, nasopharyngeal teratoma, and facial dysmorphisms. Neuroimaging revealed a duplication of the infundibula, the pituitary gland, and the dens of the epistropheus despite surgical removal of a rhino-pharyngeal mass performed at the age of two months. An array-CGH revealed a 2p12 deletion. At our evaluation, bone age assessment resulted advanced and initial pubertal activation was confirmed by Gonadotropin-Releasing Hormone stimulation test. Hormonal suppression treatment was started with satisfactory results. This case shows that DPG-plus syndrome must be considered in presence of midline and craniofacial malformations and endocrinological evaluations should be performed for the prompt and appropriate management of pubertal anomalies.

## Background

Duplication of the pituitary gland (DPG) is an extremely rare developmental anomaly (1). It may be associated with other midline malformations such as facial anomalies (median cleft lip, median cleft face syndromes, and hypertelorism), vertebral malformations, nasopharyngeal teratoma, and other central nervous system abnormalities, such as the agenesis of the corpus callosum, posterior fossa abnormalities, the absence of the olfactory bulbs and tracts, the absence of the anterior commissure, and anatomic variations of the circle of Willis. When all these anomalies are present, they are called DPG-plus syndrome (1, 2). DPG-plus syndrome is more prevalent in females and it is probably due to the splitting of the rostral notochord and prechordal plate during blastogenesis (2). DPG-plus syndrome can lead to endocrine abnormalities, mainly affecting the gonadotropic axis (3, 4). We present a case of a 5-year-old female with DPG- plus associated with midline anomalies and precocious puberty, showing how to approach differential diagnosis in presence of endocrine alterations and midline malformations.

## Case Presentation

A 5-year-old female was attended to the Pediatric Endocrinology Unit of Pediatric Clinic in Children’s Hospital of University of Parma due to onset of premature thelarche and acceleration of growth rate.

**Figure 1:**
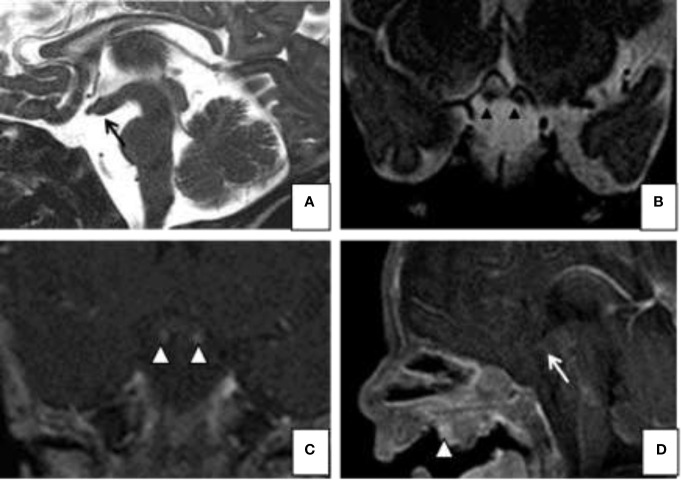
Magnetic resonance imaging (MRI). Sagittal **(A)** and coronal **(B)** T2-weighted images; Gd-enhanced coronal **(C)** T1- weighted and sagital **(D)** T1-images show on the midline sagittal plane, a thickened third ventricle floor (arrow a and d), tubomamillary fusion and the absence of a midline sella turcica and pituitary infundibulum. Coronal T2 and T1 WI MR **(C)** reveal 2 pituitary stalks (arrowheads b and c). The MRI also demonstrates a midline nasopharyngeal teratoma (arrowhead **D**).

In her medical history, it was reported that she was born by emergency caesarean delivery due to placental defect after regular full-term pregnancy, the birth weight was 2,085 g (<3° pct), the length 46 cm (10° pct), the head circumference 37 cm (50° pct). Clinical examination at birth revealed lingual hypoplasia, cleft palate, right choanal stenosis, nasopharyngeal teratoma, micrognathia, and facial dysmorphisms, with antimongolic eyelid rims and hypertelorism. At birth, magnetic resonance imaging (MRI) was obtained and showed paired infundibula extending inferiorly to two small pituitary glands, and a broad pituitary fossa (**Figure 1**). There was also a characteristic thickening of the floor of the third ventricle extending from the median eminence to the mammillary bodies. The infant’s karyotype was normal (46, XX), FISH investigation was negative, and array-CGH showed a deletion of 2p12, of uncertain clinical significance.

**Figure 2:**
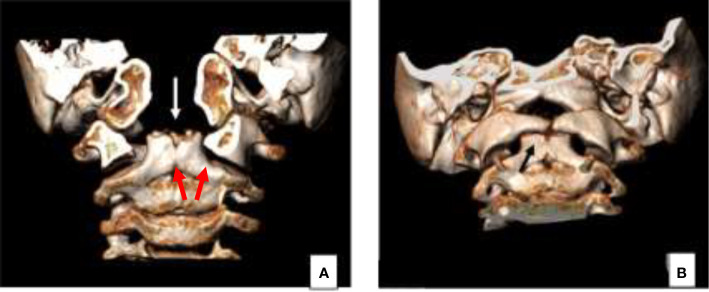
Concomitant cranio-cervical junction malformation clearly demonstrated on the 3D reconstruction CT images, characterized by duplication of the odontoid process (arrow **A, B**).

At 2 months of age, the patient underwent surgical removal of a rhino-pharyngeal mass. The mass was protruding within the oral cavity passing between the two separated palatal shelves, its approximate dimensions were 20 mm × 15 mm. The palate was characterized, in fact, by overt cleft palate, affecting both soft and hard palate. From the histopathological analysis, the mass was composed by mixed differentiated tissues of malformative origin (i.e., cutaneous and mucous epithelium, tooth germ, fibrofatty and muscular tissue, cartilage, and bone). She underwent a separate surgical procedure, at 12 months of age, with correction of hard and soft cleft palate through palatoplasty according to Sommerlad’s technique with no complications and regular recovery. A computed tomography (CT) scan without contrast of the facial skeleton was performed for the re-evaluation after surgery, which showed a duplicate appearance of the dens of the epistropheus (**Figure 2**).

The auxological parameters at 5 years of age were: height 123 cm (90° pct), weight 22.4 kg (50°–75° pct), head circumference 54 cm (>97° pct), Tanner stage P1, B3 (5, 6). Left hand and wrist X-ray was performed for bone age assessment, which showed according to Greulich and Pyle method an advanced bone age of about 3 years, corresponding to 8 years and 10 months of age (7). The patient performed Gonadotropin-Releasing Hormone (GnRH) stimulation test that showed gonadotropins values after stimulus compatible with initial pubertal activation (basal LH: 0.6 mU/ml; LH peak: 10.1 UI/ml; basal FSH: 5.0 mU/ml, FSH peak: 23.4 UI/ml); the remaining pituitary hormones were within normal limits (**Tables 1** and **2**).

**Table 1:** Results of the Gonadotropin-Releasing Hormone (GnRH) stimulation test performed at diagnosis of duplication of the pituitary gland (DPG)-plus syndrome in a 5-year-old female.

GnRH stimulation test							
**Times**	0	15’	30’	45’	60’	90’	
**LH**	0.6	6.3	10.1	10.0	9.1	7.6	mU/ml
**FSH**	5.0	12.3	15.6	20.0	23.1	23.4	mU/ml

FSH, follicle stimulating hormone; LH, luteinizing hormone.

**Table 2:** Pituitary hormones levels performed at diagnosis of duplication of the pituitary gland (DPG)-plus syndrome in a 5-year-old female.

Hormones levels	Value
**Estradiol**	< 73 pmol/L
**Progesterone**	1.2 nmol/L
**IGFBP-3**	4,770 ng/ml (+2DS/+3DS)
**Prolactin**	17.4 ng/ml
**ACTH**	17 pg/ml
**17-Hydroxyprogesterone**	0.50 ng/ml
**Cortisol**	10.5 mcg/dl

ACTH, adrenocorticotrophic hormone; IGFBP-3, Insulin-Like Growth Factor Binding Protein 3.

The child started suppression treatment with Triptorelin depot, and it is still ongoing. In particular, as current international guidelines for the treatment of Central Precocious Puberty suggest (8), she is doing intramuscular administration of 3,75 mg Triptorelin, every 28 days. After 6 months of therapy, height velocity rate was stabilized to a normal rate; after 1 year of therapy, her bone age was less accelerated (advanced of about 18 months instead of 3 years), with improvement in prediction of final height. The therapy was also effective in obtaining a partial regression and subsequent stabilization of pubertal stages evaluated according to Tanner stages.

## Discussion

DPG-plus syndrome is a rare condition with only 52 cases described worldwide and few MRI studies reported (1, 2). The rarity of this entity may hence be partially attributable to incidental detection on imaging and early mortality in some patients. Several theories have been proposed to explain the occurrence of DPG-plus syndrome and these include incomplete twinning, usually affecting the entire face, teratogens, although no specific factor has been identified, and extreme clefting (9). Whatever the injury, it is hypothesized that it occurs during blastogenesis, a process that develops during the first 4 weeks of development, after conception and before organogenesis (10). Previous reports on DPG-plus have also proposed embryologic malformations as a cause, ascribing it to a duplication of the rostral notochord and prechordal plate during early development. Defects of blastogenesis involve the formation of midline and mesoderm, leading to errors in fusion, lateralization, segmentation, morphology, and asymmetry (11).

There are no recognized genetic mutations associated with DPG-plus syndrome. Generally, the genetic control of notochord development is not well understood in humans and the genetic cause of early notochordal split during development remains unknown (10). Our patient underwent karyotype analysis at birth, which resulted normal (46, XX), FISH investigation, which was negative, and array CGH, which identified a deletion in 2p12 of about 142kB, involving a region without genes. This genomic variation was not related to the pathological phenotype of the patient, although it was not possible to definitively exclude an etiopathological involvement.

DPG-plus syndrome is usually related with multiple craniofacial anomalies like hypertelorism, cleft palate, craniopharyngeal canal, oropharyngeal teratomas, duplication of the sella, agenesis, or hypoplasia of the corpus callosum, broadening of the optic chiasma, duplication of the infundibulum, tubo-mamillary fusion, absent olfactory bulbs, duplication of the basilar artery, supernumerary teeth and duplication of the lips, tongue, and mandible (1). Our patient showed some of these features in association to aplasia of the undersurface of the tongue and bilateral optic hypoplasia. Vertebral malformations are also reported in DPG-plus syndrome, and they are commonly characterized by cleft or fusion of the vertebral bodies, rachischisis or duplication of the spinal cord, and tethered cord (9). In this regard, our patient presented a duplicate appearance of the dens of C2 vertebra.

Typical of the DPG-plus syndrome is the presence of oropharyngeal masses, of which the most frequent is teratoma, followed by dermoids cistis and polypoid masses (9). Teratomas are rare tumors arising from all three germ layers; most of them occur in the sacrococcygeal area, but they can also develop in head and neck (12). Shah et al. proposed that the development of a median cleft by a midline oropharyngeal mass led to the splitting of the pituitary anlage and the consequent generation of two pituitary glands; so, the teratoma could be the cause of the split rather than an effect of it (13). In our patient, a nasopharyngeal teratoma was noticed and surgically removed. The histological examination revealed the presence of material including a nodular formation with dental germ and covered with skin, mucosal epithelium, and multi-layered non-corneifying pavement. Epithelial islands probably belonging to the palatal cleft, cartilaginous islands, fibroadipose, and muscular tissue with edema and probably palatal bone with hematopoietic marrow were also observed.

Endocrine alterations are frequent in DPG-plus syndrome. As shown in **Table 3**, many patients, such as the child reported in this case, show precocious puberty, others may present with hypogonadotropic hypogonadism with delayed sexual development, hypothyroidism, or hyperprolactinemia DePenna et al. described two patients with precocious puberty: a 7-year-old female with a P3B3 Tanner stage and advanced bone age, and a 6.8-year-old female with thelarche, appropriate bone age and a responsive GnRH-stimulated LH level. Both had normal thyroid hormones, and prolactin levels and responded to GnRH analog treatment. In the first case, a pseudo-hamartoma was described and related to the splitting of the notochord (14). Burke et al. presented an 11-year-old patient with P4B3-4 Tanner stage, obesity with elevated fasting insulin, thyroid peroxidase antibodies and prolactin, and proposed that the dysfunction of the pituitary cells which control hormone release is a likely cause for dysregulation of the hypothalamic pituitary axis (15). Slavotinek et al. presented a case of precocious puberty with premature thelarche occurred at 2 years of age. Hormonal investigations presented a reduced level of morning cortisol, but determination of an adrenocorticotrophic hormone (ACTH) stimulation test was normal. Levels of FSH, LH, estradiol, thyroid function hormones, prolactin, insulin growth factor-1, and IGF binding protein-3 were normal (9). Vieira et al. presented a 7-year-old female with central precocious puberty related with hypertelorism, synkinesis of both hands, cervical spine anomalies with rudimentary intervertebral disks and limited neck rotation. Her Tanner Stage was B3P4 with increased basal LH and FSH levels and normal GH, thyroid stimulation hormone (TSH) and ACTH values. Thyrotropin-releasing hormone (TRH) stimulated prolactin was elevated. premature activation of the hypothalamic-pituitary-gonadal axis. Similarly to the above-mentioned cases, GnRH analog therapy was effective (16).The case described by Spiller et al. developed premature thelarche and precocious puberty at the age of four and was treated with Lupron-depot (11). More recently, Ahmed et al. reported an adult male with DPG-plus syndrome presenting with hypogonadotropic hypogonadism and delayed sexual development who underwent hormonal therapy with testosterone. Baseline values revealed a normal thyroid function (2). Although a CT of the central nervous system is the standard of care when a neonate is born with midline and craniofacial abnormalities, DPG-plus syndrome is rare and our case report is useful for future medical treatment. The etiological mechanism of the hormonal deficiency is unknown. It is hypothesized that the tubo-mammilary fusion may be a consequence of the interruption of lateral cell migration, which constitutes the hypothalamic nuclei. Therefore, the characteristic thickening of the floor of the third ventricle extending from the median eminence to the mammillary bodies, which is easily visible on midline sagittal MR image of our patient, might be related to duplication of that hypothalamic nuclei. This occurrence could explain the appearance of the endocrine alterations later in life (2).

**Table 3:** Cases of duplication of the pituitary gland (DPG)-syndrome with hypothalamic-pituitary-gonadal (HPG) axis abnormalities reported in the Literature.

Cases reported in the literature	
Precocious puberty	Case 1. de Penna et al. (14)
Precocious puberty	Case 2. de Penna et al. (14)
Precociuos puberty	Burke et al. (15)
Precocious puberty	Slavotinek et al. (9)
Delayed puberty	Ahmed et al. (2)
Precocious puberty	Vieira et al. (16)
Precocious puberty	Spiller et al. (11)

## Conclusions

DPG-plus syndrome is a plurimalformative syndrome affecting the pituitary gland, the midline and the facial structures. It is likely induced by a blastogenesis defect and can be associated with endocrine anomalies, such as precocious or delayed puberty. This case shows that DPG-plus syndrome must be considered in presence of midline and craniofacial malformations and endocrinological evaluation should be performed for the prompt and appropriate management of pubertal anomalies.

## Data Availability Statement

The raw data supporting the conclusions of this article will be made available by the authors, without undue reservation.

## Ethics Statement

The studies involving human participants were reviewed and approved by Area Vasta Emilia Romagna Nord. Written informed consent to participate in this study was provided by the participants’ legal guardian/next of kin.

## Author Contributions

GP wrote the first draft of the manuscript. MP performed the diagnosis and was in charge of the patient’s follow-up. MB, MA, and ES were in charge of the surgical patient’s management. GD and MA performed the literature review. FO performed neuroimaging exams. SE supervised the patient’s management, provided scientific contributions, and critically revised the paper. All authors contributed to the article and approved the submitted version.

## Funding

This research was supported by research grant PED-2022-01, Department of Medicine and Surgery, University of Parma, Parma, Italy.

## Conflict of Interest

The authors declare that the research was conducted in the absence of any commercial or financial relationships that could be construed as a potential conflict of interest.
